# (1*S*,3*S*)-Methyl 6,7-dimeth­oxy-1-phenyl-1,2,3,4-tetra­hydro­isoquinoline-3-carboxyl­ate

**DOI:** 10.1107/S1600536811018782

**Published:** 2011-05-25

**Authors:** Tricia Naicker, Thavendran Govender, Hendrik G. Kruger, Glenn E. M. Maguire

**Affiliations:** a School of Pharmacy and Pharmacology, University of KwaZulu Natal, Durban 4000, South Africa; b School of Chemistry, University of KwaZulu Natal, Durban 4000, South Africa

## Abstract

In the title compound, C_19_H_21_NO_4_, an organocatalyst with a tetra­hydro­isoquinoline backbone, the heterocyclic ring assumes a half-boat conformation. The dihedral angle between the aromatic rings is 82.93 (8)°. In the crystal, mol­ecules are linked *via* N—H⋯O and C—H⋯O hydrogen bonds, forming a layer parallel to (10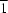).

## Related literature

For related structures, see: Naicker *et al.* (2010 ▶, 2011 ▶).
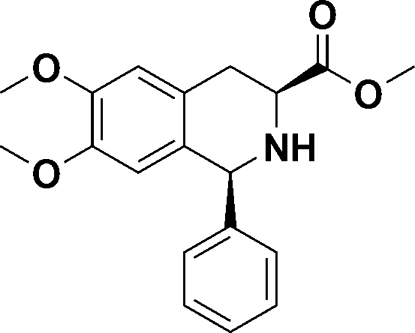


## Experimental

### 

#### Crystal data

C_19_H_21_NO_4_
*M* *_r_* = 327.37
Monoclinic, 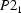

*a* = 9.3841 (3) Å
*b* = 6.3453 (2) Å
*c* = 14.2048 (4) Å
β = 94.475 (2)°
*V* = 843.25 (4) Å^3^
*Z* = 2
Mo *K*α radiation
μ = 0.09 mm^−1^
*T* = 173 K
0.90 × 0.07 × 0.06 mm

#### Data collection

Nonius KappaCCD diffractometer
Absorption correction: multi-scan (*SADABS*; Sheldrick, 1996 ▶) *T*_min_ = 0.923, *T*_max_ = 0.995
4184 measured reflections
2275 independent reflections
2138 reflections with *I* > 2σ(*I*)
*R*_int_ = 0.010

#### Refinement

*R*[*F* ^2^ > 2σ(*F* ^2^)] = 0.030
*wR*(*F* ^2^) = 0.082
*S* = 1.05
2275 reflections
222 parameters
2 restraints
H atoms treated by a mixture of independent and constrained refinement
Δρ_max_ = 0.20 e Å^−3^
Δρ_min_ = −0.13 e Å^−3^

### 

Data collection: *COLLECT* (Nonius, 2000 ▶); cell refinement: *DENZO-SMN* (Otwinowski & Minor, 1997 ▶); data reduction: *DENZO-SMN*; program(s) used to solve structure: *SHELXS97* (Sheldrick, 2008 ▶); program(s) used to refine structure: *SHELXL97* (Sheldrick, 2008 ▶); molecular graphics: *OLEX2* (Dolomanov *et al.*, 2009) ▶; software used to prepare material for publication: *SHELXL97*.

## Supplementary Material

Crystal structure: contains datablocks I, global. DOI: 10.1107/S1600536811018782/is2714sup1.cif

Structure factors: contains datablocks I. DOI: 10.1107/S1600536811018782/is2714Isup2.hkl

Additional supplementary materials:  crystallographic information; 3D view; checkCIF report

## Figures and Tables

**Table 1:** Hydrogen-bond geometry (Å, °)

*D*—H⋯*A*	*D*—H	H⋯*A*	*D*⋯*A*	*D*—H⋯*A*
N1—H1*N*⋯O3^i^	0.91 (2)	2.27 (1)	3.0918 (17)	149 (2)
C1—H1⋯O3^ii^	1.00	2.55	3.503 (2)	160
C19—H19*B*⋯O2^iii^	0.98	2.53	3.270 (2)	132

Symmetry codes: (i) 
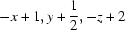
; (ii) 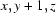; (iii) 
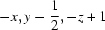
.

**Fig. 1.**
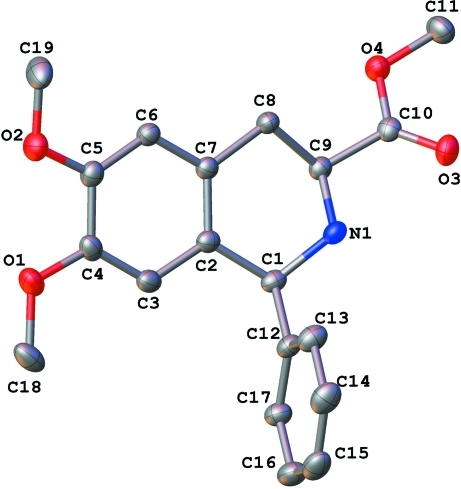
The molecular structure of the title compound with the atom numbering scheme. Displacement ellipsoids are drawn at the 40% probability level. Hydrogen atoms have been omitted for clarity.

**Fig. 2.**
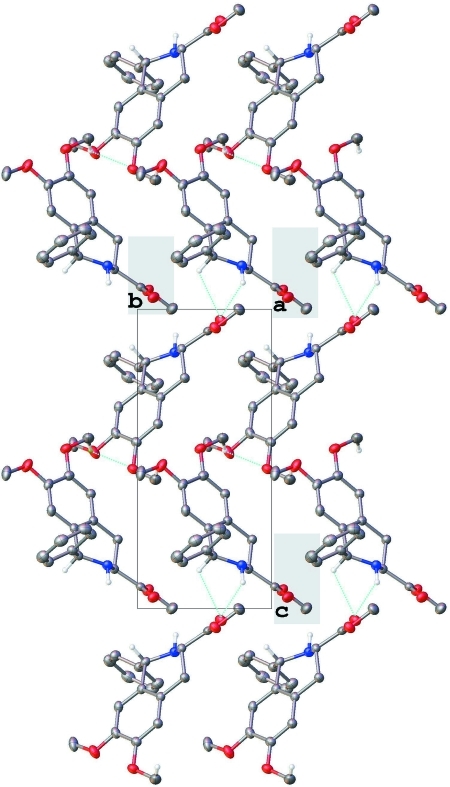
A partial projection of the title compound, viewed along the a axis.

## supplementary crystallographic information

### Comment

The title compound is a novel chiral organocatalyst containing a tetrahydroisoquinoline (TIQ) framework. We have recently reported the use of similar TIQ derivatives as organocatalysts in the Diels-Alder cycloaddition between alpha, beta-unsaturated aldehydes and cyclopentadiene (Naicker *et al.*, 2010).

Diastereomers formed during the synthesis of the title compound were easily separated using column chromatography to yield the TIQ derivative with the stereochemistry as illustrated in Fig. 1. The absolute stereochemistry was confirmed to be *S*,*S* at the C1 and C9 positions, respectively, by proton NMR spectroscopy.

The *N*-containing six-membered ring assumes a half-boat conformation [Q = 0.5537 (16) Å, θ = 53.94 (16)° and φ = 335.3 (2)°]. This observation is similar to a related structure that we recently reported (Naicker *et al.*, 2011). The molecules are linked through N1— H1N···O3^i^ and C1—H1···O3^ii^ hydrogen bonds (Table 1) into a column stacked along the *b* axis. The columns are further connected by C19—H19B···O2^iii^ hydrogen bonds, forming a layer parallel to the (101) plane (Fig. 2).

### Experimental

To a stirred solution of 1:1 methanol: methylene chloride (6.0 ml) with 4 Å molecular sieves, (*S*)-methyl 2-amino-3-(3,4-dimethoxyphenyl)propanoate (1.0 g, 4.2 mmol) and benzaldehdye (1.1 eq.) was added under an inert atmosphere. The reaction mixture was allowed to stir for 1.5 h. Thereafter the reaction mixture was filtered and the solvents was removed *in vacuo* to yield the intermediate imine which was left on a high vacuum pump to remove any residual water for 2 h. The residue was then dissolved in trifluoroacetic acid (20 ml) and refluxed for 3 h. The reaction mixture was then neutralized with a saturated sodium bicarbonate solution and extracted with ethylacetate (4 × 20 ml). The organic extracts were combined and dried over anhydrous Na_2_SO_4_ and the solvent was removed *in vacuo*. The crude product (diastereomers) was purified by column chromatography (50:50 EtOAc/Hexane, *R*_f_ 1/2) to afford the product 1.20 g (88%) as a white solid. Melting point 370–372 K. IR (neat): 2928, 2600, 1746, 1516, 1250, 1123, 727 cm^-1^ [*α*]^20^_D_ = +15.38 (*c* 0.26 in CHCl_3_) ^1^H NMR (400 MHz, CDCl_3_) δ 7.33 – 7.11 (m, 5H), 6.57 (s, 1H), 6.10 (s, 1H), 5.02 (s, 1H), 3.79 (s, 4H), 3.70 (s, 3H), 3.52 (s, 3H), 3.01 (s, 2H). ^13^C NMR (101 MHz, CDCl_3_) δ 172.96, 147.76, 147.41, 143.87, 130.22, 129.04, 128.59, 127.84, 126.07, 111.31, 110.56, 62.85, 56.54, 55.89, 55.84, 52.18, 32.22.

Recrystallization from ethyl acetate at room temperature afforded crystals suitable for X-ray analysis.

### Refinement

All hydrogen atoms, except H1N on N1, were placed in idealized positions and refined as riding, with *U*_iso_(H) = 1.2 or 1.5*U*_eq_(C). The position of H1N was located in a difference electron density map and refined with a bond length restraint of N—H = 0.95 (3) Å. With unmerged data, the Flack *x* parameter refines to -0.5475 with e.s.d. 0.6554, and the absolute structure cannot be determined reliably. The final refinements were performed with merged data.

### Crystal data

**Table:**

C_19_H_21_NO_4_	*F*(000) = 348
*M**_r_* = 327.37	*D*_x_ = 1.289 Mg m^−^^3^
Monoclinic, *P*2_1_	Melting point: 371 K
Hall symbol: P 2yb	Mo *K*α radiation, λ = 0.71073 Å
*a* = 9.3841 (3) Å	Cell parameters from 4184 reflections
*b* = 6.3453 (2) Å	θ = 2.2–28.3°
*c* = 14.2048 (4) Å	µ = 0.09 mm^−^^1^
β = 94.475 (2)°	*T* = 173 K
*V* = 843.25 (4) Å^3^	Needle, colourless
*Z* = 2	0.90 × 0.07 × 0.06 mm

### Data collection

**Table:**

Nonius KappaCCD diffractometer	2275 independent reflections
Radiation source: fine-focus sealed tube	2138 reflections with *I* > 2σ(*I*)
graphite	*R*_int_ = 0.010
φ and ω scans	θ_max_ = 28.3°, θ_min_ = 2.2°
Absorption correction: multi-scan (*SADABS*; Sheldrick, 1996)	*h* = −12→12
*T*_min_ = 0.923, *T*_max_ = 0.995	*k* = −8→8
4184 measured reflections	*l* = −18→18

### Refinement

**Table:**

Refinement on *F*^2^	Secondary atom site location: difference Fourier map
Least-squares matrix: full	Hydrogen site location: inferred from neighbouring sites
*R*[*F*^2^ > 2σ(*F*^2^)] = 0.030	H atoms treated by a mixture of independent and constrained refinement
*wR*(*F*^2^) = 0.082	*w* = 1/[σ^2^(*F*_o_^2^) + (0.0478*P*)^2^ + 0.1004*P*] where *P* = (*F*_o_^2^ + 2*F*_c_^2^)/3
*S* = 1.05	(Δ/σ)_max_ < 0.001
2275 reflections	Δρ_max_ = 0.20 e Å^−^^3^
222 parameters	Δρ_min_ = −0.13 e Å^−^^3^
2 restraints	Extinction correction: *SHELXL97* (Sheldrick, 2008), Fc^*^=kFc[1+0.001xFc^2^λ^3^/sin(2θ)]^-1/4^
Primary atom site location: structure-invariant direct methods	Extinction coefficient: 0.014 (4)

### Special details

**Table:**

Experimental. Half sphere of data collected using *COLLECT* strategy (Nonius, 2000). Crystal to detector distance = 33 mm; combination of φ and ω scans of 1.0°, 60 s per °, 2 iterations.
Geometry. All e.s.d.'s (except the e.s.d. in the dihedral angle between two l.s. planes) are estimated using the full covariance matrix. The cell e.s.d.'s are taken into account individually in the estimation of e.s.d.'s in distances, angles and torsion angles; correlations between e.s.d.'s in cell parameters are only used when they are defined by crystal symmetry. An approximate (isotropic) treatment of cell e.s.d.'s is used for estimating e.s.d.'s involving l.s. planes.
Refinement. Refinement of *F*^2^ against ALL reflections. The weighted *R*-factor *wR* and goodness of fit *S* are based on *F*^2^, conventional *R*-factors *R* are based on *F*, with *F* set to zero for negative *F*^2^. The threshold expression of *F*^2^ > σ(*F*^2^) is used only for calculating *R*-factors(gt) *etc*. and is not relevant to the choice of reflections for refinement. *R*-factors based on *F*^2^ are statistically about twice as large as those based on *F*, and *R*- factors based on ALL data will be even larger.

### Fractional atomic coordinates and isotropic or equivalent isotropic displacement parameters (Å^2^)

**Table:**

	*x*	*y*	*z*	*U*_iso_*/*U*_eq_	
O1	0.32761 (13)	0.8193 (2)	0.52227 (9)	0.0421 (3)	
O2	0.14691 (13)	0.5304 (2)	0.46573 (8)	0.0377 (3)	
O3	0.39059 (11)	−0.1210 (2)	0.96117 (8)	0.0372 (3)	
O4	0.15273 (11)	−0.0802 (2)	0.93763 (8)	0.0359 (3)	
N1	0.44475 (13)	0.2219 (2)	0.85516 (9)	0.0286 (3)	
H1N	0.4969 (18)	0.214 (4)	0.9118 (11)	0.033 (5)*	
C1	0.45806 (15)	0.4396 (3)	0.82212 (10)	0.0276 (3)	
H1	0.4271	0.5382	0.8715	0.033*	
C2	0.36095 (14)	0.4684 (2)	0.73185 (10)	0.0260 (3)	
C3	0.38575 (16)	0.6398 (3)	0.67243 (11)	0.0303 (3)	
H3	0.4558	0.7420	0.6921	0.036*	
C4	0.30923 (16)	0.6610 (3)	0.58571 (11)	0.0309 (3)	
C5	0.20784 (15)	0.5077 (3)	0.55602 (10)	0.0299 (3)	
C6	0.17693 (14)	0.3466 (3)	0.61690 (10)	0.0273 (3)	
H6	0.1036	0.2485	0.5984	0.033*	
C7	0.25316 (14)	0.3266 (2)	0.70613 (10)	0.0250 (3)	
C8	0.21706 (15)	0.1462 (3)	0.76978 (10)	0.0274 (3)	
H8A	0.2446	0.0110	0.7415	0.033*	
H8B	0.1127	0.1433	0.7759	0.033*	
C9	0.29613 (15)	0.1715 (3)	0.86760 (10)	0.0259 (3)	
H9	0.2524	0.2909	0.9011	0.031*	
C10	0.28876 (15)	−0.0258 (3)	0.92677 (10)	0.0273 (3)	
C11	0.1344 (2)	−0.2585 (3)	0.99879 (13)	0.0423 (4)	
H11A	0.0322	−0.2859	1.0025	0.063*	
H11B	0.1788	−0.2277	1.0621	0.063*	
H11C	0.1799	−0.3829	0.9733	0.063*	
C12	0.61296 (15)	0.4843 (3)	0.80537 (10)	0.0291 (3)	
C13	0.68782 (16)	0.3487 (3)	0.74980 (11)	0.0374 (4)	
H13	0.6419	0.2270	0.7229	0.045*	
C14	0.82974 (17)	0.3906 (4)	0.73342 (12)	0.0426 (4)	
H14	0.8798	0.2980	0.6951	0.051*	
C15	0.89763 (17)	0.5663 (4)	0.77280 (13)	0.0431 (4)	
H15	0.9951	0.5924	0.7631	0.052*	
C16	0.82347 (18)	0.7041 (3)	0.82631 (13)	0.0419 (4)	
H16	0.8695	0.8269	0.8520	0.050*	
C17	0.68109 (16)	0.6639 (3)	0.84278 (11)	0.0339 (3)	
H17	0.6307	0.7594	0.8796	0.041*	
C18	0.43336 (19)	0.9743 (3)	0.54802 (15)	0.0450 (4)	
H18A	0.4362	1.0782	0.4972	0.067*	
H18B	0.5270	0.9062	0.5585	0.067*	
H18C	0.4097	1.0450	0.6061	0.067*	
C19	0.0553 (2)	0.3649 (3)	0.43094 (12)	0.0480 (5)	
H19A	0.0173	0.3981	0.3664	0.072*	
H19B	−0.0238	0.3498	0.4715	0.072*	
H19C	0.1094	0.2328	0.4308	0.072*	

### Atomic displacement parameters (Å^2^)

**Table:**

	*U*^11^	*U*^22^	*U*^33^	*U*^12^	*U*^13^	*U*^23^
O1	0.0381 (6)	0.0362 (7)	0.0523 (7)	−0.0028 (6)	0.0043 (5)	0.0163 (6)
O2	0.0426 (6)	0.0405 (7)	0.0296 (5)	−0.0005 (6)	−0.0009 (4)	0.0067 (5)
O3	0.0353 (6)	0.0404 (7)	0.0356 (6)	0.0093 (6)	0.0002 (4)	0.0060 (5)
O4	0.0313 (5)	0.0380 (7)	0.0380 (6)	−0.0025 (5)	−0.0002 (4)	0.0088 (5)
N1	0.0229 (6)	0.0322 (7)	0.0299 (6)	0.0000 (5)	−0.0032 (5)	0.0002 (6)
C1	0.0234 (6)	0.0302 (8)	0.0290 (7)	−0.0003 (6)	0.0007 (5)	−0.0056 (6)
C2	0.0214 (6)	0.0254 (7)	0.0315 (7)	0.0018 (6)	0.0031 (5)	−0.0026 (6)
C3	0.0237 (6)	0.0251 (7)	0.0422 (8)	0.0006 (6)	0.0023 (5)	−0.0008 (7)
C4	0.0263 (6)	0.0281 (8)	0.0390 (8)	0.0036 (6)	0.0077 (6)	0.0067 (7)
C5	0.0266 (7)	0.0337 (8)	0.0296 (7)	0.0040 (6)	0.0030 (5)	0.0018 (6)
C6	0.0237 (6)	0.0286 (7)	0.0296 (7)	0.0000 (6)	0.0012 (5)	−0.0001 (6)
C7	0.0218 (6)	0.0257 (7)	0.0276 (6)	0.0017 (6)	0.0027 (5)	−0.0004 (6)
C8	0.0243 (6)	0.0284 (7)	0.0289 (7)	−0.0023 (6)	−0.0012 (5)	0.0021 (6)
C9	0.0240 (6)	0.0271 (7)	0.0267 (6)	0.0021 (6)	0.0017 (5)	−0.0016 (6)
C10	0.0302 (7)	0.0291 (8)	0.0223 (6)	0.0015 (6)	0.0008 (5)	−0.0040 (6)
C11	0.0477 (9)	0.0388 (10)	0.0404 (9)	−0.0081 (8)	0.0035 (7)	0.0082 (8)
C12	0.0232 (6)	0.0350 (8)	0.0284 (7)	−0.0005 (6)	−0.0017 (5)	−0.0012 (6)
C13	0.0290 (7)	0.0449 (10)	0.0381 (8)	0.0022 (8)	0.0010 (6)	−0.0077 (8)
C14	0.0299 (7)	0.0590 (12)	0.0394 (8)	0.0068 (8)	0.0068 (6)	−0.0014 (9)
C15	0.0247 (7)	0.0602 (12)	0.0445 (9)	−0.0010 (8)	0.0029 (6)	0.0106 (9)
C16	0.0314 (8)	0.0473 (11)	0.0460 (9)	−0.0093 (8)	−0.0031 (7)	0.0041 (9)
C17	0.0286 (7)	0.0370 (9)	0.0357 (8)	−0.0045 (7)	0.0002 (6)	−0.0014 (7)
C18	0.0414 (9)	0.0287 (9)	0.0673 (12)	−0.0008 (8)	0.0203 (8)	0.0072 (9)
C19	0.0671 (12)	0.0411 (11)	0.0331 (8)	0.0001 (10)	−0.0126 (8)	0.0006 (8)

### Geometric parameters (Å, °)

**Table:**

O1—C4	1.3696 (19)	C8—H8B	0.9900
O1—C18	1.424 (2)	C9—C10	1.512 (2)
O2—C5	1.3704 (18)	C9—H9	1.0000
O2—C19	1.421 (2)	C11—H11A	0.9800
O3—C10	1.2017 (18)	C11—H11B	0.9800
O4—C10	1.3429 (18)	C11—H11C	0.9800
O4—C11	1.445 (2)	C12—C17	1.392 (2)
N1—C9	1.4552 (18)	C12—C13	1.394 (2)
N1—C1	1.467 (2)	C13—C14	1.395 (2)
N1—H1N	0.909 (14)	C13—H13	0.9500
C1—C12	1.5178 (19)	C14—C15	1.380 (3)
C1—C2	1.525 (2)	C14—H14	0.9500
C1—H1	1.0000	C15—C16	1.382 (3)
C2—C7	1.381 (2)	C15—H15	0.9500
C2—C3	1.407 (2)	C16—C17	1.398 (2)
C3—C4	1.383 (2)	C16—H16	0.9500
C3—H3	0.9500	C17—H17	0.9500
C4—C5	1.403 (2)	C18—H18A	0.9800
C5—C6	1.384 (2)	C18—H18B	0.9800
C6—C7	1.4118 (18)	C18—H18C	0.9800
C6—H6	0.9500	C19—H19A	0.9800
C7—C8	1.513 (2)	C19—H19B	0.9800
C8—C9	1.5317 (19)	C19—H19C	0.9800
C8—H8A	0.9900		
			
C4—O1—C18	117.28 (14)	C8—C9—H9	108.9
C5—O2—C19	116.41 (13)	O3—C10—O4	123.84 (15)
C10—O4—C11	115.40 (13)	O3—C10—C9	124.95 (14)
C9—N1—C1	110.63 (12)	O4—C10—C9	111.20 (12)
C9—N1—H1N	109.5 (12)	O4—C11—H11A	109.5
C1—N1—H1N	106.5 (15)	O4—C11—H11B	109.5
N1—C1—C12	109.42 (13)	H11A—C11—H11B	109.5
N1—C1—C2	108.75 (12)	O4—C11—H11C	109.5
C12—C1—C2	111.26 (12)	H11A—C11—H11C	109.5
N1—C1—H1	109.1	H11B—C11—H11C	109.5
C12—C1—H1	109.1	C17—C12—C13	119.00 (14)
C2—C1—H1	109.1	C17—C12—C1	120.67 (14)
C7—C2—C3	119.79 (13)	C13—C12—C1	120.31 (14)
C7—C2—C1	121.43 (13)	C12—C13—C14	120.43 (18)
C3—C2—C1	118.75 (13)	C12—C13—H13	119.8
C4—C3—C2	120.72 (14)	C14—C13—H13	119.8
C4—C3—H3	119.6	C15—C14—C13	120.18 (18)
C2—C3—H3	119.6	C15—C14—H14	119.9
O1—C4—C3	125.04 (15)	C13—C14—H14	119.9
O1—C4—C5	115.36 (14)	C14—C15—C16	119.84 (16)
C3—C4—C5	119.55 (14)	C14—C15—H15	120.1
O2—C5—C6	124.75 (14)	C16—C15—H15	120.1
O2—C5—C4	115.61 (14)	C15—C16—C17	120.37 (18)
C6—C5—C4	119.64 (13)	C15—C16—H16	119.8
C5—C6—C7	120.76 (14)	C17—C16—H16	119.8
C5—C6—H6	119.6	C12—C17—C16	120.14 (16)
C7—C6—H6	119.6	C12—C17—H17	119.9
C2—C7—C6	119.20 (13)	C16—C17—H17	119.9
C2—C7—C8	121.88 (12)	O1—C18—H18A	109.5
C6—C7—C8	118.89 (13)	O1—C18—H18B	109.5
C7—C8—C9	110.34 (12)	H18A—C18—H18B	109.5
C7—C8—H8A	109.6	O1—C18—H18C	109.5
C9—C8—H8A	109.6	H18A—C18—H18C	109.5
C7—C8—H8B	109.6	H18B—C18—H18C	109.5
C9—C8—H8B	109.6	O2—C19—H19A	109.5
H8A—C8—H8B	108.1	O2—C19—H19B	109.5
N1—C9—C10	109.60 (12)	H19A—C19—H19B	109.5
N1—C9—C8	108.29 (11)	O2—C19—H19C	109.5
C10—C9—C8	112.19 (12)	H19A—C19—H19C	109.5
N1—C9—H9	108.9	H19B—C19—H19C	109.5
C10—C9—H9	108.9		
			
C9—N1—C1—C12	−177.34 (12)	C5—C6—C7—C8	−179.14 (14)
C9—N1—C1—C2	−55.63 (14)	C2—C7—C8—C9	9.86 (19)
N1—C1—C2—C7	16.42 (18)	C6—C7—C8—C9	−171.91 (13)
C12—C1—C2—C7	137.00 (14)	C1—N1—C9—C10	−163.99 (11)
N1—C1—C2—C3	−161.44 (13)	C1—N1—C9—C8	73.33 (15)
C12—C1—C2—C3	−40.86 (19)	C7—C8—C9—N1	−46.80 (16)
C7—C2—C3—C4	−4.0 (2)	C7—C8—C9—C10	−167.89 (11)
C1—C2—C3—C4	173.89 (14)	C11—O4—C10—O3	3.2 (2)
C18—O1—C4—C3	−0.3 (2)	C11—O4—C10—C9	−175.80 (13)
C18—O1—C4—C5	−177.71 (14)	N1—C9—C10—O3	2.7 (2)
C2—C3—C4—O1	−178.59 (14)	C8—C9—C10—O3	123.00 (16)
C2—C3—C4—C5	−1.2 (2)	N1—C9—C10—O4	−178.38 (12)
C19—O2—C5—C6	−5.8 (2)	C8—C9—C10—O4	−58.05 (15)
C19—O2—C5—C4	173.61 (15)	N1—C1—C12—C17	−130.54 (15)
O1—C4—C5—O2	3.6 (2)	C2—C1—C12—C17	109.27 (16)
C3—C4—C5—O2	−174.04 (14)	N1—C1—C12—C13	51.37 (18)
O1—C4—C5—C6	−177.03 (13)	C2—C1—C12—C13	−68.82 (19)
C3—C4—C5—C6	5.4 (2)	C17—C12—C13—C14	1.2 (3)
O2—C5—C6—C7	175.00 (14)	C1—C12—C13—C14	179.29 (16)
C4—C5—C6—C7	−4.4 (2)	C12—C13—C14—C15	0.5 (3)
C3—C2—C7—C6	5.0 (2)	C13—C14—C15—C16	−1.9 (3)
C1—C2—C7—C6	−172.82 (13)	C14—C15—C16—C17	1.6 (3)
C3—C2—C7—C8	−176.77 (13)	C13—C12—C17—C16	−1.4 (2)
C1—C2—C7—C8	5.4 (2)	C1—C12—C17—C16	−179.55 (15)
C5—C6—C7—C2	−0.9 (2)	C15—C16—C17—C12	0.1 (3)

### Hydrogen-bond geometry (Å, °)

**Table:**

*D*—H···*A*	*D*—H	H···*A*	*D*···*A*	*D*—H···*A*
N1—H1N···O3^i^	0.91 (2)	2.27 (1)	3.0918 (17)	149 (2)
C1—H1···O3^ii^	1.00	2.55	3.503 (2)	160
C19—H19B···O2^iii^	0.98	2.53	3.270 (2)	132

Symmetry codes: (i) −*x*+1, *y*+1/2, −*z*+2; (ii) *x*, *y*+1, *z*; (iii) −*x*, *y*−1/2, −*z*+1.
